# Anti-*Trimeresurus albolabris* venom IgY antibodies: preparation, purification and neutralization efficacy

**DOI:** 10.1186/s40409-016-0078-3

**Published:** 2016-08-24

**Authors:** Hai-long Duan, Qi-yi He, Bin Zhou, Wen-wen Wang, Bo Li, Ying-zheng Zhang, Qiu-ping Deng, Ying-feng Zhang, Xiao-dong Yu

**Affiliations:** 1 Animal Toxin Group, Chongqing Key Laboratory of Animal Biology, Chongqing Engineering Research Center of Bioactive Substance, Engineering Research Center of Active Substance and Biotechnology, Ministry of Education, College of Life Science, Chongqing Normal University, Chongqing, 401331 China; 2 Library, Chongqing Normal University, Chongqing, 401331 China

**Keywords:** IgY, Egg yolk, Snake venom, *Trimeresurus albolabris*, LD_50_

## Abstract

**Background:**

Snakebite incidence in southwestern China is mainly attributed to one of the several venomous snakes found in the country, the white-lipped green pit viper *Trimeresurus albolabris*. Since antivenom produced from horses may cause numerous clinical side effects, the present study was conducted aiming to develop an alternative antivenom antibody (immunoglobulin Y - IgY) from leghorn chickens.

**Methods:**

IgY in egg yolk from white leghorn chicken previously injected with *T. albolabris* venom was extracted by water, precipitated by ammonium sulfate and purified by affinity chromatographic system*.* IgY was identified by SDS-PAGE, ELISA and Western blot, and its neutralizing assay was conducted on mice.

**Results:**

Chickens injected multiple times with *T. albolabris* venom elicited strong antibody responses, and from their egg yolk IgY was isolated and purified, which exhibited a single protein band on SDS-PAGE and two bands (about 65 and 35 kDa, respectively) under reduced conditions. Immunoblot analysis revealed that these IgY are polyclonal antibodies since they bind with most venom components. In the neutralizing assay, all mice survived while the ratios of IgY/venom reached up to 3.79 (50.0 mg/13.2 mg).

**Conclusions:**

IgY antibody response was successfully conducted in white leghorn chicken injected with *T. albolabris* venom. IgY against *T. albolabris* venom was obtained for the first time, and it exhibited strong neutralizing potency on mice. These results may lay a foundation for the development of IgY antivenom with clinical applications in the future.

## Background

Snakebite, a neglected tropical disease, comprises an important worldwide public health concern, particularly in the rural areas of tropical regions such as Africa, Asia and Latin America [1, 2]. In southwestern China, the frequent snakebite cases in rural regions are mainly attributable to *Trimeresurus albolabris, Naja naja* and *Deinagkistrodon acutus*. In these areas, antivenom raised in horses are recommended for the treatment of most snakebite cases. However, snake antivenom may provoke numerous side effects such as anaphylactic shock, pyrogen reaction, serum sickness and skin rash [3]. In addition, the preparation and purification of antivenom from horse blood is laborious, time-consuming, expensive and low yielding [4–7].

To overcome the disadvantages of antivenom made from horse blood, it is essential to attempt other pathways to produce the antibodies against snake venom. In this study, we employed chickens to produce antivenom antibodies. Chicken egg yolk antibody (immunoglobulin Y - IgY) has been identified as a potential, convenient, non-invasive and inexpensive source of polyclonal antibodies [8, 9]. The advantages of IgY are well documented in the literature [10–12]. Currently, the preparations of IgY from chicken egg yolk have been successfully made by immunizing chickens with venoms of several venomous snake species [13–18]. For the first time, we used venom of the white-lipped green pit viper (*T. albolabris*) from southwestern China to immunize white leghorn chickens and attempt to prepare and purify IgY, and further to assess its neutralization efficacy.

## Methods

### Reagents

Freund’s complete adjuvant (FCA), incomplete Freund’s adjuvant (IFA), Tween-20, ortho-phenylenediamine (OPD) and 3,3’-diaminobenzidine tetrahydrochloride (DAB) were purchased from Sigma (USA). Peroxidase conjugated rabbit anti-chicken IgY was obtained from Earthox (USA). All other reagents were of analytical grade, purchased from local companies.

### Animals

Seventeen-week-old white leghorn hens weighing 1.5 kg each, purchased from a local poultry farm, in good health and laying conditions (laying 5–6 eggs per week) were used for the production of IgY against snake venom. They were kept in individual cages with standard food and water. Kunming mice (21–23 g each), obtained from the Laboratory Animal Center of the Third Military Medical University, were used to determine venom lethality and its neutralization.

### Venom

Venoms were milked from *T. albolabris* captured in Guangxi province, China. Venom was lyophilized in a ModulyoD-230 freeze dryer (Thermo Scientific, USA) and stored at −20 °C until required.

### Determination of LD_50_

Snake venom (0.5 mL in saline) was intraperitoneally (IP) injected into Kunming mice. Mice were divided into six groups of ten each and IP received the doses (1.82, 2.30, 2.87, 3.58, 4.48 and 5.60 mg/kg). The results were recorded after 72 h and LD_50_ was calculated according to the method described by Bliss [19] and expressed as microgram (μg) per mouse.

### Immunization assay

For the initial immunization, four hens were immunized intramuscularly at multiple sites in their chest with 0.5 mL saline (containing 329 μg of snake venom) emulsified with an equal volume of FCA. On the 15th and 35th day after the first immunization, hens received booster doses of 0.5 mL saline (containing 658 and 1316 μg of snake venom, respectively) emulsified with an equal volume of IFA. Eggs were collected daily from the first day to the 84th day after the first immunization, individually identified, and stored at 4 °C until required.

### Isolation and purification of IgY

The eggs obtained from the 21th to 84th day after the first immunization were used to isolate IgY that was obtained from yolk according to the modified method of Akita et al. [20]. Briefly, the yolk, separated from the egg white, was diluted ten-fold with cold distilled water and adjusted a final pH of up to 5.2 with 0.1 N HCl under stirring. The yolk suspensions were stored overnight at 4 °C. The supernatants containing the IgY, the water-soluble fraction (WSF), were collected by centrifugation at 10,000 × *g* for 30 min at 4 °C and were subjected to 35 % saturated ammonium sulfate solution for precipitation. The salt pellet was collected by centrifugation at 10,000 × *g* for 30 min at 4 °C, the precipitated proteins were dissolved in 0.02 M phosphate buffer (PBS, pH 7.5, containing 0.6 M sodium sulfate) and dialyzed against the same solution.

For further purification, isolated IgY was loaded on to the HiTrap IgY Purification HP column (Amersham, Sweden) equilibrated with 0.02 M PBS, pH 7.5, containing 0.6 M sodium sulfate and according to the Amersham’s product instruction. Then, the fractions were pooled, dialyzed against PBS and stored at −20 °C until further use. The purity and titer of the preparations were determined by SDS-PAGE and ELISA, respectively, and their protein concentrations were determined by Lowry’s method [21].

### ELISA assay

The optimal dilution of antibodies was determined by ELISA according to Voller et al. [22]. Briefly, polystyrene 96-well microtiter plates (Corning, USA) were coated with 5 μg/mL snake venom in a coating buffer (0.1 M carbonate bicarbonate, pH 9.6) for 12 h at 4 °C. The wells were washed six times with washing buffer (PBS, pH 7.4, containing 0.05 % Tween-20). The wells were blocked for 2 h at 37 °C with a blocking buffer (3 % BSA in washing buffer). The wells were washed again three times with washing buffer. Serial dilutions of IgY samples in blocking buffer were prepared and 100 μL of each diluted IgY sample was added into individual coated wells and the plates were incubated at 37 °C for 1.5 h. The wells were washed five times with the same washing buffer.

The plates were incubated with peroxidase conjugated rabbit anti-chicken IgY (1:5000) for 45 min at 37 °C. After the plates were washed five times, 100 μL of substrate buffer (0.1 M citric acid, plus 0.2 M sodium diphosphate, 5.0 mL H_2_O, 5.0 mg OPD, 5 mL of H_2_O_2_) were added and incubated at room temperature in the dark for 20 min. The reaction was stopped by addition of 50 μL of 2 N sulfuric acid. Absorbance was read at 490 nm on an ELISA plate reader (Molecular Devices Corporation, USA). IgY samples from the eggs collected before immunization were used as negative control. Wells free of venom were used as blanks.

### Western blot assay

Western blot was carried out based on the modified procedure of Towbin et al. [23]. In brief, 12.5, 25 and 50 μg of venom were mixed with SDS-PAGE sample buffer, heated for 3 min at 100 °C under non-reducing conditions, then separated on 10 % SDS-PAGE gel [24]. Proteins on the gel were electroblotted for 2 h at 100 V onto PVDF membranes in Mini Trans-Blot system (Bio-Rad, USA). After blotting, the membranes were blocked with blocking buffer (PBS, pH 7.4, with 0.5 % Tween-20 and 2 % non-fat dry milk) for 1 h at room temperature, then washed with washing buffer (0.01 M PBS, pH 7.4, 0.05 % Tween-20). Blots were then incubated with diluted anti-venom IgY (1:1000) in the same blocking buffer on a horizontal shaker at room temperature overnight. After six washes, blots were incubated with peroxidase conjugated rabbit anti-chicken IgY diluted 1:5000 in blocking buffer at room temperature for 2 h. After three washes, blots were incubated in peroxidase chromogenic substrate solution (0.01 M PBS, pH 7.4 with 0.05 % DAB and 0.03 % H_2_O_2_) for 20 s. Finally, the immunoreactivity was stopped with distilled water.

### Neutralization studies

To evaluate the neutralization effect of IgY, the challenge dose of *T. albolabris* venom was four times larger than the LD_50_ dose. Purified IgY at different concentrations was mixed with the challenge dose of venom. The mixtures were incubated at 37 °C for 30 min, and then 0.5 mL was IP injected into four groups of ten mice for each dose. The control group received the same amount of venom dissolved in the same volume of saline solution with negative IgY. The survival was determined 72 h later.

## Results

### Antibody response

**Fig. 1:**
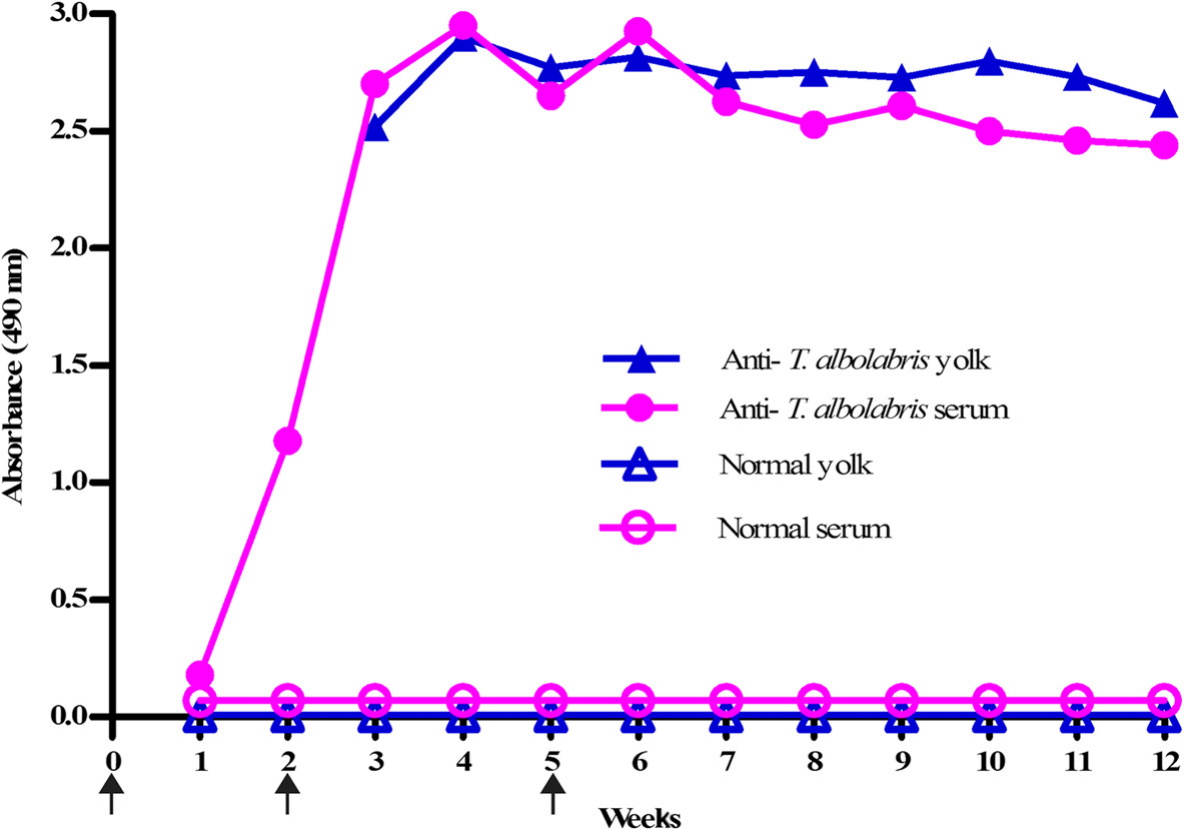
Primary and secondary antibody response in serum and egg yolk of chickens immunized with *T. albolabris* venom. Anti-*T. albolabris* (WSF, water-soluble fraction) activity was determined by ELISA (as described in Materials and Methods section). Arrows indicate immunization

To evaluate IgY response in the immunization process, the titer of IgY was carried out by ELISA. Following primary immunization, the chickens elicited comparable antibody response, which was detected in serum by day 7. There was a sharp increase in antibody titer both in serum and egg yolk, although the pre-booster response was very low. This secondary response was maintained thereafter by the second booster injection (Fig. 1).

### Isolation and specific activity of IgY

**Fig. 2:**
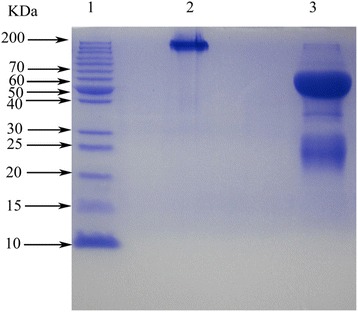
SDS-PAGE analysis (10 % resolving gel) of anti-*T. albolabris* IgY. Lanes: (1) molecular weight marker; (2) non-reduced IgY; and (3) reduced IgY

**Fig. 3:**
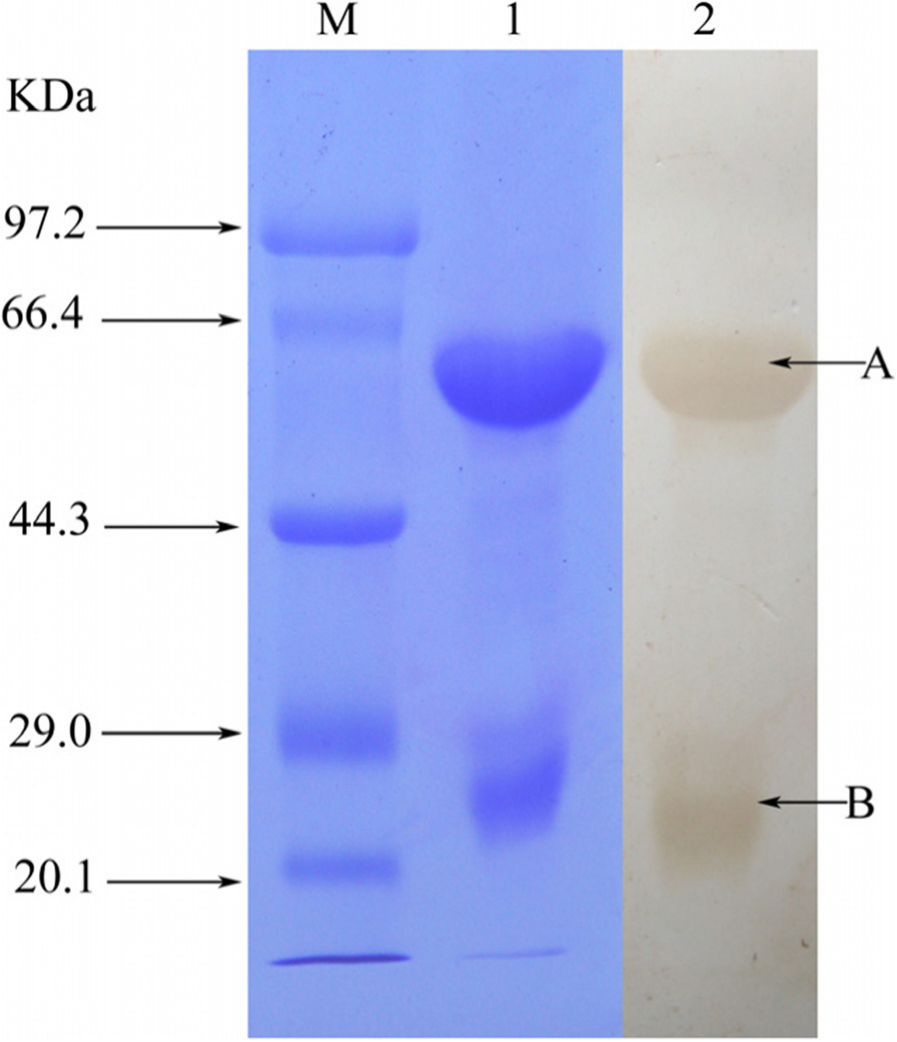
Identification of anti-*T. albolabris* venom IgY. The reduced IgY (50 μg) was separated on 10 % SDS-PAGE and stained with Coomassie brilliant blue (lane M: molecular weight marker; lane 1: reduced IgY). Result of Western blot test using peroxidase conjugated rabbit anti-chicken IgY to detect the heavy chains (lane 2: A) and the light chains (lane 2: B)

After three steps (water dilution, ammonium sulfate precipitation and thiophilic chromatography), IgY from egg yolk was obtained and exhibited a single protein band (180 ~ 200 kDa) through SDS-PAGE. Under reduced conditions, it presented two bands of about 65 kDa and 35 kDa, respectively (Fig. 2), which represented one heavy chain and one light chain of IgY demonstrated by Western blot analysis (Fig. 3). The average recovery of venom-specific IgY from 127 eggs was about 18.43 % (Table 1). The venom-specific activities of IgY in the different fractions, including water-soluble fraction (WSF), salting-out fraction (SOF) and thiophilic-chromatography fraction (TCF), were compared by ELISA, and the results showed that the titer of TCF is twofold higher than SOF, and 16 times higher when compared with to WSF. Obviously, the three purification steps used resulted in the enrichment of venom-specific IgY.

**Table 1:** Recovery ratio of proteins and titer of IgY fractions

Fractions	Titer of IgY by ELISA (×10^4^)	Yolk proteins in 127 eggs (g)	Recovery ratio of proteins (%)
WSF	3.2	31.79	100
SOF	25.6	12.53	39.41
TCF	51.2	5.86	18.43

*WSF* water-soluble fraction, *SOF* salting-out fraction, *TCF* thiophilic-chromatography fraction

### Western blot

**Fig. 4:**
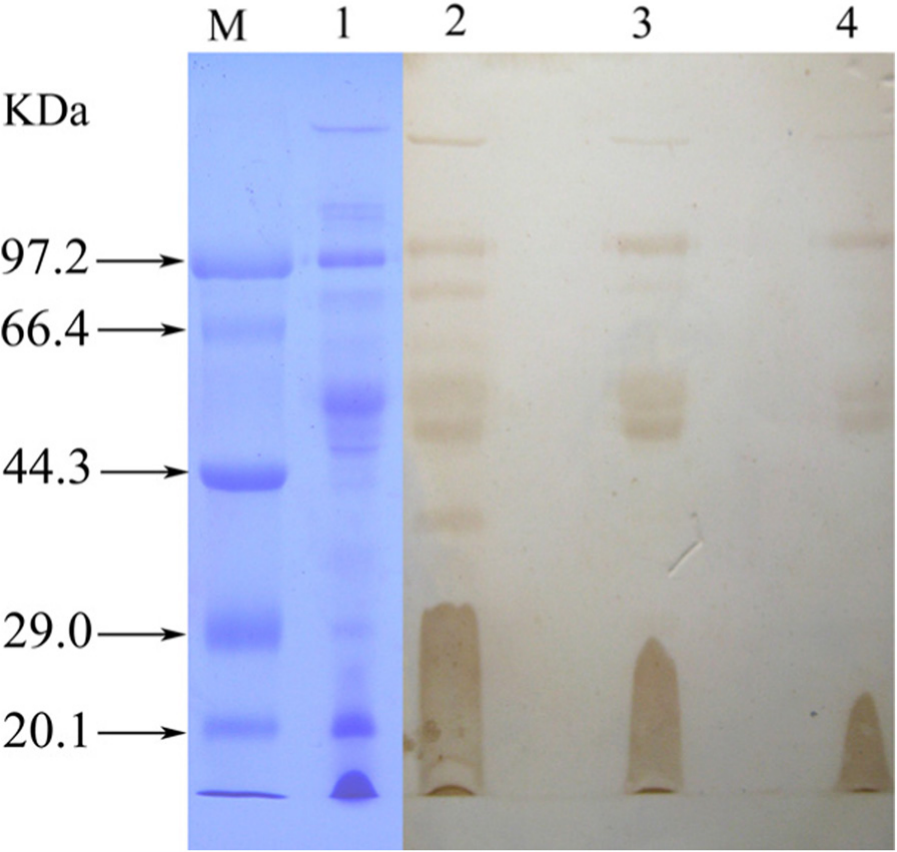
Anti-*T. albolabris* venom IgY combined with venom proteins by Western blot analysis. Lane 1: 50 μg of venom on 10 % SDS-PAGE; lane 2: 50 μg of venom; lane 3: 25 μg of venom; and lane 4: 12.5 μg of venom combined with a fixed amount of anti-*T. albolabris* venom IgY (1:1000)

Different amounts of crude venom (50, 25 and 12.5 μg) were subjected to SDS-PAGE, electroblotted onto a PVDF membrane, and finally incubated with an enough amount of IgY antivenom (1:1000). The results revealed that IgY bound to venom protein components on PVDF membrane in a dose-dependent manner (Fig. 4).

### Lethality of venom and neutralization effect

The LD_50_ of *T. albolabris* venom is 3.3 mg/kg of mouse body weight. All mice were dead within 2 h after injection of 4 LD_50_ (13.2 mg/kg, defined as challenge dose). When the different amounts of purified IgY (20.0 ~ 50.0 mg/kg) were added to the venom challenge dose, the neutralizing ability was evident in a dose-response manner, and when IgY/venom ratios reached up to 3.79 (50.0 mg/13.2 mg) all animals survived without noticeable toxic reactions.

## Discussion

Usually, snake antivenom is produced by injecting horses or sheep with small doses of the venom. The standard procedure for immunization of horses for commercial antivenom production requires at least ten injections given in ten weeks for the first course, followed by two more courses [25]. The use of chickens as substitutes of horses or sheep for antibody production brings a number of advantages; the most evident is the non-invasive, rapid and economical manner of producing antibodies [26]. Compared to the production in horses or sheep, the IgY technology presents several benefits: no bleeding, only egg collection is required on immunization; IgY isolation is fast and simple; and very low quantities of antigen are required to obtain high and long-lasting IgY titers in the yolk from immunized hens [27].

Initially, IgY antibodies were thought of as IgG-like immunoglobulins [28]. However, there are important differences between IgY and IgG regarding their function, including: IgY does not bind the rheumatoid factor (RF) whereas IgG molecules often cause false positive results by interaction with RF in immunoassays; IgY do not interfere with mammalian IgG and they do not activate the mammalian complement system [29–31]. Previous studies have shown that hens immunized with snake venoms produced IgY that could neutralize the toxic and lethal effects of venoms and could serve to treat domestic animals envenomed by snake [13–18]. The current study for the first time describes the creation of anti-*T. albolabris* venom antibody in chicken egg yolk and its efficacy in neutralizing the lethal effects of *T. albolabris* venom.

In the current study, the antibody production in IgY egg yolk started 15 days after the first injection of *T. albolabris* venom, increasing progressively along the immunization procedure, and attaining a plateau after the second booster, which was maintained thereafter (Fig. 1). Anti-*T. albolabris* venom antibody response in chicken proceeded like the antibody response of other snake venoms reported [17, 18, 25].

In the literature, it has been reported that high pure IgY could be obtained through three principal steps [16–18, 25]. Isolation of total IgY in the egg yolk was carried out under controlled conditions including low pH and low ionic concentration of acidified water, so that the aggregation of yolk lipids and separation of IgY in the clear supernatant occurred. The lipophilic solvents were used to separate yolk lipids from soluble proteins, but the purity and activity of the preparations by the lipophilic solvent method was lower than by the water dilution method [32]. After precipitation of solid ammonium sulfate and thiophilic chromatography, the TCF exhibited a single band (180 ~ 200 kDa) under native state and two bands (about 68 and 25 kDa) under reduced condition on SDS-PAGE (Fig. 2), which indicated the purity of obtained IgY. Additionally, the reduced TCF was combined with polyclonal rabbit anti-chick IgY by Western blot analysis (Fig. 3), which demonstrated the TCF’s IgY activity. IgY activity in TCF was found to be two and 16 times higher than its corresponding activity in SOF and WSF, respectively. Obviously, as described in previous studies, the three steps resulted in the enrichment of venom-specific IgY [11, 20, 33]. The average recovery of venom-specific IgY from 127 eggs was about 18.43 % and 5.86 g pure IgY was obtained (Table 1).

It has been confirmed by Western blot analysis that the anti-*T. albolabris* IgY was able to recognize and bind to most protein components of *T. albolabris* venom (Fig. 4). This suggests that IgY is a specific anti-*T. albolabris* venom polyclonal antibody. A mixture of IgY and venom was injected into mice and its neutralizing ability was obvious in dose-dependent manner.

## Conclusion

In conclusion, for the first time an IgY antivenom was successfully raised through immunizing hens with *T. albolabris* venom and had strong neutralizing ability against the snake venom. The results may lay a foundation for therapy of snakebites. However, further studies are essential to confirm the safety and efficacy of such finding as an antidote to human victims of snakebites.
